# Higher Incidence of Cancer Therapy-Related Cardiac Dysfunction in the COVID-19 Era: A Single Cardio-Oncology Center Experience

**DOI:** 10.3390/jcdd10010023

**Published:** 2023-01-06

**Authors:** Daniela Di Lisi, Cristina Madaudo, Luca Di Fazio, Antonino Gulotta, Oreste Fabio Triolo, Alfredo Ruggero Galassi, Lorena Incorvaia, Antonio Russo, Giuseppina Novo

**Affiliations:** 1 Division of Cardiology, University Hospital Paolo Giaccone, 90127 Palermo, Italy; 2 Department of Health Promotion, Mother and Child Care, Internal Medicine and Medical Specialties (PROMISE) “G. D’Alessandro”, University of Palermo, 90127 Palermo, Italy; 3 Department of Surgical, Oncological and Oral Sciences, University of Palermo, Via del Vespro 129, 90127 Palermo, Italy

**Keywords:** cardio-oncology, cardiotoxicity, COVID-19, pandemic, CTRCD

## Abstract

Aim: COVID-19 pandemic had a big impact on our life, it has revolutionized the practice of cardiology and the organization of hospital and outpatient activities. Thus the aim of our study was to assess the impact of the COVID-19 pandemic on the development of cancer therapy-related cardiac dysfunction (CTRCD). Methods and results: A single center retrospective study was carried out evaluating 96 cancer patients treated with anthracyclines and admitted to our Cardio-Oncology unit from June to August 2019 and 60 patients from June to August 2021. The incidence of CTRCD was assessed performing an echocardiogram at the time of the enrollment. We found a significantly higher incidence of CTRCD in the second period compared to first period (13% vs. 2%, *p* value 0.0058). In addition we found that fewer yearly visits were performed in our Cardio-oncology unit in 2021 compared to 2019 (300 patients/year in 2019 vs. 144 patients/year in the COVID era). Conclusion: COVID-19 pandemic seems to influence the onset of CTRCD in cancer patients by indirectly reducing hospital access of cancer patients and cardiological checks. In addition our data reflect the impact of the COVID-19 pandemic in the late diagnosis of cancer, in the reduction of hospital admissions and regular medical checks, in the increase of comorbidities and cardiovascular complications.

## 1. Introduction

Anticancer drugs can cause several cardiovascular complications such as arrhythmias, arterial hypertension, pulmonary hypertension, myocardial ischemia, thrombovascular events, left ventricular dysfunction and heart failure [1,2,3].

2022 ESC Guidelines on cardio-oncology introduced the definition “cancer therapy-related cardiovascular toxicity” (CTR-CVT) to include all cardiovascular toxicity effects of anticancer-drugs. For cardiac injury, cardiomyopathy, and heart failure, the descriptive term cancer therapy-related cardiac dysfunction (CTRCD) is recommended [4].

CTRCD is a common complication of anticancer drugs, especially during treatment with high doses of anthracyclines [5].

Instead thrombovascular events are mainly caused by tyrosine kinase inhibitors, vascular endothelial growth factor inhibitors. Especially anti BCR-ABL can cause accelerated atherosclerosis, myocardial infarction, peripheral arterial disease [6].

2020 ESMO consensus recommendations defined CTRCD as an absolute decrease in the left ventricular ejection fraction (LVEF) of >20 percentage points or an absolute decrease in the LVEF of 10 percentage points to a value of <50% or an absolute decrease in the LVEF to a value of <50% [7].

Subclinical cardiac dysfunction was defined as an absolute decrease from baseline in the left ventricular global longitudinal strain (GLS) of 5% or a relative decrease from baseline in the GLS of 12% or troponins elevation from baseline [7].

2022 ESC Guidelines on cardio-oncology defined aymptomatic CTRCD mild as a relative decline in GLS by >15% from baseline AND/OR new rise in cardiac biomarkers [4].

Cardiac biomarkers (troponin and natriuretic peptides-NP) should be measured in patients at risk of CTRCD where available and results should be interpreted according to the patient clinical status, type of cancer treatment, and kidney function.

In the past, Cardinale et al. showed that troponin was able to identify patients at risk of future cardiotoxicity, if treated with high dose of anthracyclines [8].

Nevertheless there is a lot of evidence that cardiac biomarkers cannot predict cardiotoxicity of anticancer agents as well. The predictive value of cardiac biomarkers (especially NP) still needs exploration.

Over the years various strategies have been proposed in the Cardio-Oncology field in order to identify the early signs of cardiotoxicity during anticancer treatment [9,10]. 

For example, several studies showed the usefulness of GLS alone or combined with high- sensitivity troponin to predict future cardiotoxicity in women with breast cancer [11,12].

Today the role of the GLS is consolidated in the diagnosis of subclinical cardiac dysfunction during chemotherapy; other imaging modalities such as cardiac magnetic resonance [13,14] and new echocardiographic parameters are being studied in the early diagnosis of cardiotoxicity [15].

Recently, the Cardio-Oncology Study Group of the Heart Failure Association (HFA) of the European Society of Cardiology (ESC) in collaboration with the International Cardio-Oncology Society (ICOS) proposed a baseline cardiovascular risk stratification proformas that can be used by oncology and haemato-oncology teams to stratify cancer patients for cardiovascular risk before initiation of potentially cardiotoxic cancer therapies [16].

This risk stratification model is very important because it allows medical teams to devise the best surveillance protocol for patients on the basis of their baseline cardiovascular risk and chemotherapy used.

The proposed surveillance protocols are based on the monitoring of LVEF, GLS and biomarkers. A more intensified surveillance protocol with closer medical checks is envisaged for high and very high risk patients [17].

Unfortunately, the COVID-19 pandemic has impacted us, revolutionizing the practice of cardiology and the organization of hospital and outpatient activities.

The risk of COVID-19 infection reduced patients access to hospitals, cardioncological and other medical checks and for this reason the use of telemedicine has grown in the field of cardiology and Cardio-Oncology. New surveillance protocols have been proposed for monitoring cardiotoxicity in cancer patients during the COVID-19 pandemic [18,19]. 

The monitoring of Cardiotoxicity was carried out through fewer hospital visits, fewer check-ups, and telemedicine: echocardiography was expected in high-risk patients and in patients with symptoms and signs of heart failure, and it was postponed in asymptomatic and low-risk patients during chemotherapy; also, a baseline cardiological evaluation was not performed in low-risk patients [18,19]. Calvillo-Argüelles et al. also suggested performing focused echocardiographic studies during the COVID-19 pandemic because the primary question in cancer patients is the left ventricular function, and short protocols to assess left ventricular function with a focus on two-dimensional imaging may be sufficient; they also suggested avoiding ECG leads because ECG cables are challenging to clean between patients and may become a source of COVID-19 transmission [18].

The impact of the pandemic on ischemic heart disease is already known. For example, the pandemic has led to increased incidence of mechanical complications resulting from late presentation of patients with ST segment elevation myocardial infarction [20]. Little is known about the impact of the pandemic on the development of CTRCD. Thus, the aim of our study was to assess the impact of the COVID-19 pandemic on the development of CTRCD, showing the experience of a single Cardio-Oncology centre.

## 2. Methods 

A single centre retrospective study was carried out evaluating patients admitted to Cardio-Oncology unit after the third wave of the COVID-19 pandemic (from June to August 2021) compared to patients admitted during a 2 year period prior to the COVID-19 pandemic (from June to August 2019). 300 patients/year in 2019 vs. 144 patients/year in the COVID era (2021) performed a cardiological visit in our Cardio-Oncology unit. 

Inclusion criteria were: patients with hematological (lymphoma) or solid tumor (breast cancer) referred to our Cardio-Oncology unit for baseline cardiological examination or follow-up; current or previous (<3 months) treatment with anthracyclines and/or anti HER2 (human epidermal receptor 2); minimum cumulative dose of ≥250 mg/mq of doxorubicin or epirubicin > 125 mg/mq. 

Exclusion criteria were: pre-existing heart failure with mildly reduced or reduced LVEF, previous myocardial infarction or other heart diseases causing a reduction in LVEF, severe valve diseases. From June to August 2019, in our Cardio-Oncology unit, 96 patients underwent cardiological clinical and instrumental assessment blood pressure measurement, an electrocardiogram with QTc interval measurement and a complete Color-Doppler echocardiogram with speckle tracking echocardiography (STE) and myocardial deformation indices measurement. 

Echocardiographic evaluation was carried out using a GE Vivid E95 ultrasound system prime echocardiography machine (GE Healthcare, Chicago, IL, USA) and a 4Vc-D (1.4–5.2 MHz) linear transducer (GE Healthcare, Chicago, IL, USA). An assessment of the cardiac chamber dimensions, an evaluation of the systolic and diastolic ventricular function was carried out according to the ASE/EACVI recommendations [21,22]. Myocardial deformation indices of left ventricle (GLS) were measured in all patients using GE software (Echopac V.202, GE Healthcare, Chicago, IL, USA), with STE method, before the COVID-19 pandemic.

During baseline cardiological visit and subsequent cardiological checks, the patient’s cardiovascular risk profile was optimized and treatment with angiotensin-converting enzyme inhibitor (ACEI) or angiotensin receptor blocker (ARB) or beta blockers were started in patients with arterial hypertension, statin was started in patients with dyslipidemia; smoking cessation was recommended in patients who smoked. 

In addition, in this first period, patients performed baseline cardiological evaluations or cardiological check-ups after previous cardiological checks scheduled on the basis of a rigorous surveillance protocol that included not only cardiological visits with electrocardiogram but also echocardiography every 3 months in all patients and every 2 cycles in patients at high risk of cardiotoxicity.

From June to August 2021, 60 patients were referred to our Cardio-Oncology ambulatory for baseline cardiological evaluation or follow-up visit. Unfortunately, due to the COVID-19 pandemic, many patients who came for a cardiological visit after starting chemotherapy had never performed a baseline cardiological evaluation in our ambulatory (they had performed only an electrocardiogram before starting chemotherapy or focus echocardiogram without GLS measurement); in addition many patients had not followed a standard surveillance protocol in the previous months of the pandemic.

In this second period, Cardio-Oncology visits were performed by vaccinated physicians; both operators and patients wore FFP2 masks. Considering the risk of COVID-19 transmission, in this second period, we performed a focused echocardiogram assessing the main questions such as right and left ventricular function, the presence of pericardial effusion. We avoided using ECG leads in some patients to reduce the risk of COVID-19 transmission and we did not perform GLS analysis in all patients but only in 50% of patients, especially new patients performing baseline cardiological evaluation before starting chemotherapy.

On the other hand, the other patients who had started chemotherapy during the pandemic did not have a baseline GLS to use as a comparison. In addtion protocols of chemotherapy were comparable between 2019 and 2021; cardiological examination was performed by the same group of specialist in our department; echocardiogram was performed with the same machine.

Quantitative variables were reported as mean and standard deviation; the differences between the analyzed groups were studied with the two-tailed Student *t*-test for independent samples. Qualitative variables were reported as a percentage. Chi-square test was used for the comparison of percentages. A *p* value < 0.05 was considered statistically significant. Qualitative variables were reported as a percentage. The analysis was performed using MedCalc^®^ software (MedCalc Software, Mariakerke, Belgium).

## 3. Results

A 156 patients were referred to our Cardio-Oncology unit during two different periods (before the COVID-19 pandemic and after the third wave of the COVID-19 pandemic) were enrolled. Of which, 96 patients (49 women and 47 men, median age 52 ± 9.4 years old, BMI 24 ± 3.9 Kg/mq) were enrolled during the first period and 60 patients (39 women and 21 men, median age 65 ± 5 years old, BMI 25 ± 5 Kg/mq) were enrolled during the second period. The characteristics of the study population are summarized in Table 1.

We analyzed cardiovascular risk factors of patients, previous chemotherapy or radiotherapy, type of tumor, median duration of chemotherapy, time of the cardiological visit at the time of the enrollment (baseline or follow-up).

During the first period, 21% of the enrolled patients had performed previous chemotherapy and 21% previous radiotherapy. In the second period, 25% of patients had performed previous chemotherapy and 16% previous radiotherapy. In addition, hematological tumor was present in 40% of patients assessed before the pandemic and in 50% of patients assessed after the third wave pandemic. Solid tumor was present respectively in 60% and 50% of patients. 

At the time of the enrollment, 59% of patients were undergoing a follow-up visit after a standard and rigorous surveillance protocol during the first period; during the second period 50% of patients were undergoing a follow-up visit without a previous check-up or baseline cardiological evaluation including echocardiography. Other patients enrolled performed baseline cardiological evaluation before starting chemotherapy. The median duration of chemotherapy in patients assessed during follow-up check-ups was 12 ± 4 months in the first period and 12 ± 2 months in the second period. All patients were treated with antrhacyclines. In addition, in the second period, at least 50% of the patients had received a first dose of the SARS-CoV-2 vaccine without notable symptoms. 45% of the population had SARS-CoV-2 infection.

Analyzing the two different populations (population assessed before the pandemic and population assessed after the third wave of the pandemic), we did not find significant differences regarding the general characteristics of the population. Only median age of the population was significantly higher in patients evaluated during COVID-19 pandemic compared to population evaluated before COVID-19 pandemic (*p* value < 0.0001). We did not find a significant difference regarding the incidence of cardiovascular risk factors, the type of tumor and the number of baseline visits between the two periods (Table 1). Respectively, 15% and 16% of patients were treated with ACEI or ARB in the two periods; 5% and 8% of patients were treated with betablockers respectively in the first and second period.

In addition, we didn’t find significant changes in electrocardiographic parameters in all patients assessed at baseline and during follow-up. We didn’t find a significant increase in Qtc interval, we found no ventricular or supraventricular arrhythmias. 

Unfortunately, we have no consistent data on NT pro BNP values or troponins. Prior to the pandemic, sampling was performed only in 20% of patients in order not to expose patients to repeated sampling. Also, after the third wave COVID-19 pandemic, NT-pro BNP and troponins were assessed only in few patients and thus the data are not consistent. 

Echocardiographic examination with GLS measurement was performed in all patients during the first period at baseline and every 3 months; during the second period we performed echocardiography with GLS measurement only in few patients assessed for the first time considering that patients assessed during follow-up do not have baseline GLS. In fact, during the COVID-19 pandemic some patients performed focus echocardiographic study without GLS measurement and without ECG leads to reduce the risk of COVID-19 transmission; echocardiography was performed every 6 months or 12 months in the COVID-19 era. See Table 2.

Thus, we assessed the incidence of CTRCD in both periods. The incidence of subclinical cardiac dysfuction such as reduction in GLS in according with ESMO guidelines, was assessed only during the first period.

During the first period, 38% of patients assessed developed sublcinical cardiac dysfunction. We didn’t find other cardiovascular events in cancer patients in the two periods analyzed. 

**Figure 1:**
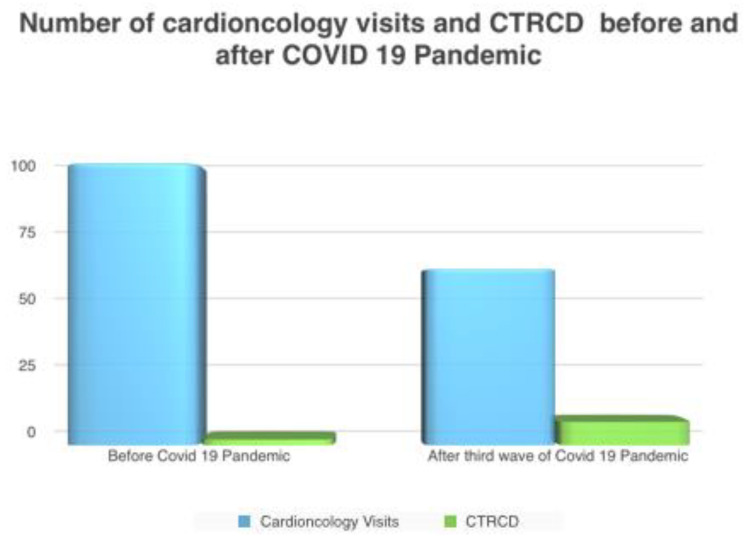
Number of Cardio-Oncology visits and CTRCD before and after COVID-19 Pandemic.

**Figure 2:**
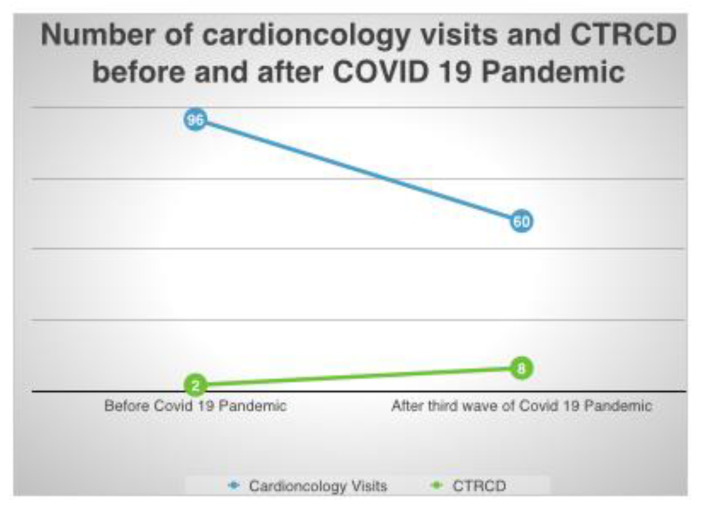
Graphical rapresentation of CTRCD and Cardio-Oncology visits before and after COVID-19 Pandemic.

In analyzing the incidence of CTRCD, we found a significant increase of CTRCD after the third wave of the COVID-19 pandemic compared to the same period before the COVID-19 pandemic (13% vs. 2%, *p* value 0.0058) (Figure 1 and Figure 2).

Patients that developed CTRCD before the COVID-19 pandemic were 2 patients (a 70 years old women and a 75 years old women) with metastatic breast cancer treated with previous radiotherapy (5 years earlier), subsequently high doses of anthracyclines, trastuzumab and taxol for another year.

Analyzing 8 patients that developed CTRCD after the third wave COVID-19 pandemic we observed that:

- 7 patients (4 patients with breast cancer and 3 patients with lymphoma) developed CTRCD earlier during anticancer treatment with anthracyclines after a median period of 1 year; these patients have not performed previous radiotherapy or chemotherapy; their median age was 65 ± 10 years old; 3 patients had arterial hypertension and 1 patient had diabetes as cardiovascular risk factors; only electrocardiogram and focus echocardiogram without GLS measurement were performed before starting chemotherapy in these patients.
- only 1 patient (a 68 years old women with arterial hypertension) with breast cancer and previous chemotherapy with high doses of anthracyclines, had late cardiotoxicity developing CTRCD 2 years after the end of the treatment.
- 1 patient with lymphoma (a 72 year old man), who developed early CTRCD after anthracycline treatment was hospitalized for COVID-19 pneumonia during the second wave; 4 patients who developed CTRCD had asymptomatic COVID-19 infection and were isolated.

## 4. Discussion

Our data reflects the experience of a single Italian Cardio-Oncology centre showing the impact of the COVID-19 pandemic causing increased cardiotoxicity in cancer patients.

A higher incidence of CTRCD was found in our Cardio-Oncology unit after the third wave of COVID-19 pandemic compared to the same period in 2019 before the COVID-19 pandemic. 

Patients analyzed before the COVID-19 pandemic had the same general characteristics of the patients analyzed after the third wave COVID-19 pandemic; the same type of tumor and the same chemotherapy treatment with antracyclines. They had no significant differences regarding cardiovascular risk factors or the duration of cancer treatment. Despite the absence of significant differences regarding factors affecting a greater risk of cardiotoxicity between the 2 populations, a higher incidence of CTRCD was found after the third wave COVID-19 pandemic, indirectly showing the effects of the pandemic. 

In fact, during the first period analyzed, two cases of late onset cardiotoxicity were found in patients with previous radiotherapy and high dose of anthracyclines and thus in patients at higher risk of cardiotoxicity. Before the COVID-19 pandemic, patients underwent standard and rigorous surveillance protocols, echocardiography was performed every 3 months during treatment with anthracyclines or every two cycles in patients at higher risk, or every year after the end of chemotherapy, in accordance with previous and current guidelines [5,6,7]. The cardiovascular risk profile was optimized before starting chemotherapy. Patients were subjected to more complete and closer checks. 

After the third wave COVID-19 pandemic, we found more cases of CTRCD (1 case of late onset cardiotoxicity in a patient with arterial hypertension and previous treatment with high dose of anthracyclines at high risk of cardiotoxicity).

Surprising was the higher incidence of early CTRCD in 7 patients without previous chemotherapy and/or radiotherapy or previous cardiovascular diseases and therefore at lower risk of cardiotoxicity.

Due to the pandemic and the risk of COVID-19 transmission these patients had not undergone a standard surveillance protocol; they had performed an electrocardiogram and focus echocardiogram without GLS measurement before starting chemotherapy and then at 1 year without performing frequent/regular cardiological checks due to the COVID-19 pandemic or careful optimization of cardiovascular risk factors.

Thus, we assumed that the execution of less regular/frequent cardiological checks during chemotherapy, a focus echocardiogram during the pandemic without GLS measurement only in high-risk or symptomatic patients as well as the reduced access of patients to the hospital due to the risk of infection, the lack of a guided GLS approach contributed to the greater development of cardiotoxicity.

Rules and guidelines changed between 2019 and 2022. On the basis of 2022 ESC guidelines on cardioncology, the patient surveillance protocol should be planned on the basis of the baseline cardiovascular toxicity risk; echocardiogram after anthracyclines is recommended at baseline and every 2 cycles in high and very high-risk patients; echocardiography should be considered after a cumulative dose of 250 mg/mq of doxorubicin or equivalent in moderate-risk patients; echocardiography may be considered in low-risk patients (IIb) after a cumulative dose of 250 mq/mq of doxorubicin or equivalent.

In all adults receiving anthracycline chemotherapy, an echocardiogram is recommended before starting chemotherapy and within 12 months after completing treatment. 

In patients receiving neoadjuvant or adjuvant HER2-targeted therapies, echocardiography is recommended every 3 months and within 12 months after completing treatment. In low-risk HER2+ EBC patients who are asymptomatic and with a normal assessment after 3 months, reducing monitoring to every 4 months may be considered. In high- and very high-risk HER2+ EBC patients, more frequent echocardiography monitoring should be considered during treatment. Troponin and NT-pro BNP monitoring before every cycle during anthracycline chemotherapy and 3 and 12 months after therapy completion is recommended in high- and very high-risk patients. Cardiology referral is recommended in high-risk and very high-risk patients before anticancer therapy. In patients categorized at moderate CV toxicity risk, cardiology referral may be considered. It is recommended that patients categorized to be at low CV toxicity risk should proceed to anticancer therapy without delay. Discussion of the risk/benefit balance of cardiotoxic anticancer treatment in high- and very high-risk patients in a multidisciplinary approach prior to starting treatment is recommended [4]. Therefore 2022 ESC guidelines underline the importance of more intensive monitoring in patients at high and very high risk of CV toxicity; in all adults receiving anthracycline chemotherapy, an echocardiogram is recommended before starting chemotherapy and within 12 months after completing treatment.

In our study, in 2019, all patients treated with anthracyclines received intensive follow-up every 3 months. In COVID-19 era follow-up was not performed regularly and cardioprotective measures were implemented to a lesser extent.

In addition, the annual number of cardio-Oncology visits performed in 2021 was lower than in 2019. The number of patients that had received therapy in 2021 was half of the patients treated in 2019. These data are very important, they reflect the impact of the COVID-19 pandemic in the late diagnosis of cancer, in the reduction of hospital admissions and regular medical checks, in the increase of comorbidities and cardiovascular complications. Furthermore our study showed that population in 2021 was significantly older.

It is known that elderly patients have a greater risk of developing cardiotoxicity especially patients >65 years. So certainly older age in association with less monitoring during the COVID-19 pandemic may have contributed to the increased risk of developing CTRCD.

In fact before anthracyclines chemotherapy, age between 65 and 79 years old represents a risk factor “medium 2” and age ≥80 years represents a “high” risk factor using the HFA/ICOS risk stratification tool [16]. Probably being older during COVID-19 pandemic, patients would have benefited more from a closer follow-up, as suggested by the 2022 ESC guidelines on cardioncology [4].

In addition, 5 patients that developed early CTRCD had COVID-19 infection (1 patient COVID-19 pneumonia and other patients asymptomatic infection). 

Thus, we hypothesized that COVID-19 infection could influence and favor the onset of early cardiotoxicity in patients treated with anthracyclines, either directly by inducing myocardial damage or indirectly by reducing regularly scheduled hospital check-ups of cancer patients. In fact, the COVID-19 infection contributed to the reduction in outpatient visits and hospital follow-ups delaying administration of therapies (both chemotherapy and cardioprotective drugs).

In addition, it is known that the COVID-19 infection is often accompanied by myocardial damage, myocarditis, thrombosis and other cardiovascular complications [23].

In fact, COVID-19 can cause myocardial injury and myocardial infarction with several mechanisms. For example, COVID-19 may lead to an increased propensity for plaque disruption and thrombus formation [24] leading to type 1 myocardial infarction; increased cardiometabolic demand associated with the systemic infection or sepsis coupled with hypoxia caused by acute respiratory illness can impair myocardial oxygen demand-supply relationship and lead to additional myocardial injury. 

Another potential mechanism leading to acute non-ischaemic myocardial injury is direct injury by COVID-19 through ACE2 receptors, which are present in the myocardium and are functional receptors for COVID-19. Patients with heart failure have a higher expression of ACE2, which may explain their increased risk for myocardial injury following COVID-19 [25].

Therefore to reduce hospital admissions and infections, follow-up visits in cancer patients were drastically reduced during COVID-19 pandemic, also considering that the cardiologists were employed in the COVID wards. Thus only urgent cardiology visits in symptomatic cancer patients were performed during first wave COVID-19 pandemic. Probably the reduction of surveillance in the COVID era influenced the subsequent greater access of patients with cardiotoxicity to our Cardio-Oncology center after the third wave COVID-19. Our observational study want to underline the sharp impact of the COVID-19 pandemic in the hospital organization and in the health field. In recent years COVID-19 has turned life around the world upside down.

## 5. Conclusions

The COVID-19 pandemic seems to influence and favor the onset of CTRCD in patients treated with anthracyclines; indirectly reducing access of cancer patients to the hospital and performing less regular/frequent check-ups and focus echocardiography without GLS measurement during chemotherapy, directly through myocardial damage induced by COVID-19. 

Our data are preliminary and needs to be confirmed by other studies.

Certainly, our study wants to underline the importance of an adequate cardiological follow-up in cancer patients during and after chemotherapy in order to prevent cardiotoxicity, also during the COVID-19 pandemic.

## 6. Study Limitations

First, this study is monocentric, retrospective and the population is heterogeneous.

Second, we did not perform blood chemistry tests or cardiac magnetic resonance to demonstrate a direct link between heart damage and COVID-19.

Therefore, our conclusions are not supported by objective data but simply describe what we have observed in our cardio-Oncology center.

Certainly, our study wants to underline the importance of adequate follow-up in cancer patients based on their risk of cardiotoxicity, showing the impact of COVID-19 pandemic. 

It is certainly recommended to resume regularly scheduled follow-ups in cancer patients as soon as possible, also during the COVID-19 pandemic, ensuring adequate protection from SARS-CoV-2 infection.

It cannot be denied that COVID-19 has revolutionized clinical cardiology practice, but adequate monitoring in Cardio-Oncology is necessary to prevent cardiotoxicity and to start cardioprotective drugs.

## Figures and Tables

**Table 1:** Patients’ characteristics.

	Before COVID-19 Pandemic (June–August 2019)	During COVID-19 Pandemic (June–August 2021)	*p* Value
Outpatient visits with transthoracic echocardiography	96	60	
Median age (years old)	52 ± 9.4	65 ± 5	*p* < 0.0001
n. female (%)	49 (52%)	39 (66%)	0.08
BMI, kg/m^2^	24 ± 3.9	25 ± 5	0.16
Arterial hypertension n° pts (%)	19 (20%)	13 (22%)	0.76
Dyslipidemia n° pts (%)	24 (25%)	15 (25%)	1
Diabetes n° pts (%)	17 (18%)	13 (22%)	0.54
Smoke n° pts (%)	52 (55%)	32 (54%)	0.9
ACEI or ARB treatment	14 (15%)	10 (16%)	0.86
Beta blockers	5 (5%)	5 (8%)	0.45
Statin	24 (25%)	15 (25%)	1
Solid tumor n° (%)	58 (60%)	30 (50%)	0.22
Hematological tumor n° (%)	38 (40%)	30 (50%)	0.22
Previous chemotherapy	20 (21%)	15 (25%)	0.56
Previous radiotherapy	20 (21%)	10 (16%)	0.44
Baseline visit n° pts (%)	40 (41%)	30 (50%)	0.27
Follow-up visits n° pts (%)	56 (59%)	30 (50%)	0.27
Median duration of chemotherapy treatment (months ± *DS*)	12 ± 4	12 ± 2	1
Chemotherapy-related cardiac dysfunction (n° pts)	2 (2%)	8 (13%)	0.0058

**Table 2:** Cardiological follow-up in pre COVID-19 era and in the COVID-19 Era.

	Pre COVID-19		COVID-19 Era	
	Baseline	Follow-Up	Baseline	Follow-Up
Cardiological evaluation	yes	every 3 months	no	no
Electrocardiogram	yes	every 3 months	yes	every 6 months
Echocardiogram	Yes with ECG monitoring and GLS Measurement	every 3 months	Yeswithout GLS measurement and without ECG monitoring to reduce exposition time and contact	every 6 months or 12 months if clinical stability
Nt pro BNP, Troponin measurement	yes	every 3 months	no	no

## Data Availability

Not applicable.
